# 999. Nasal Mucosal Cytokines: Potential Biomarkers for Pediatric Pneumonia Severity and Etiology

**DOI:** 10.1093/ofid/ofab466.1193

**Published:** 2021-12-04

**Authors:** Rouba Sayegh, Ki Wook Yun, Zhaohui Xu, Rebecca Wallihan, Sarah Marzec, Amy Leber, Kathy Everhart, Daniel M Cohen, Ankita P Desai, Sherman J Alter, Lilliam Ambroggio, Todd A Florin, Meghan Keaton, Sara Mertz, Samir S Shah, Richard Ruddy, Desiree Jones, Osama El-assal, Asuncion Mejias, Octavio Ramilo

**Affiliations:** 1 Nationwide Children’s Hospital, Columbus, Ohio; 2 Seoul National University College of medicine, Seoul; 3 Nationwide Children’s Hospital, Columbus, OH; 4 University Hospitals/Rainbow Babies and Children’s Hospital, Cleveland, OH; 5 Dayton Children’s Hospital, Dayton, OH; 6 Children’s Hospital Colorado, Denver, CO; 7 Ann & Robert H Lurie Children’s Hospital of Chicago, Chicago, IL; 8 Promedica Toledo Children’s Hospital, Toledo, OH; 9 Cincinnati Children’s Hospital Medical Center, Cincinnati, OH; 10 University of Cincinnati College of Medicine, Cincinnati, Ohio; 11 Akron Children’s Hospital, Akron, Ohio

## Abstract

**Background:**

Community acquired pneumonia (CAP) is a leading cause of mortality in children < 5 years, but our understanding of disease pathogenesis remains limited. The objective of this study was to define the local host immune response in the respiratory tract by measuring nasal mucosal cytokine (NMC) concentrations (conc.). We hypothesized that NMC represent a potential biomarker to help assessing disease severity and pathogen classification.

**Methods:**

We leveraged nasopharyngeal (NP) samples and clinical data from an observational multicenter study [Children’s Hospital’s Initiative for Research in Pneumonia (CHIRP)] conducted between 2015 and 2018. We measured conc. of 92 NMC using the Olink immunoassay. NMC conc. were compared by severity-defined by need for hospitalization, mild (outpatient) and severe (inpatient), and by identified pathogen using Mann-Whitney U test.

**Results:**

This substudy included 182 children with CAP (mild=61; severe=121) and 30 healthy controls (HC). The pathogens identified included: 101 viruses; 32 bacteria (pyogenic=10; atypical=22); 12 with >1 pathogen; and 37 with no pathogen. Children with severe CAP had greater CCL23 and MCP-3 conc. than those with mild disease (p=0.012; p=0.011 respectively). When comparing NMC profiles of children with CAP of viral and bacterial etiology, the viral group had greater conc. of proinflammatory cytokines IL-6 and TNF, (p=0.0002; p=0.0098 respectively). Further subgroup analysis showed that CAP secondary to influenza virus had greater conc. of IL-6, TNF, and antiviral INF-γ and IP-10 compared with CAP caused by pyogenic bacteria. IL-6 and MCP1-4 were significantly increased in the influenza group compared to the atypical bacteria group.

Quantification of NMC in children with CAP based on disease severity

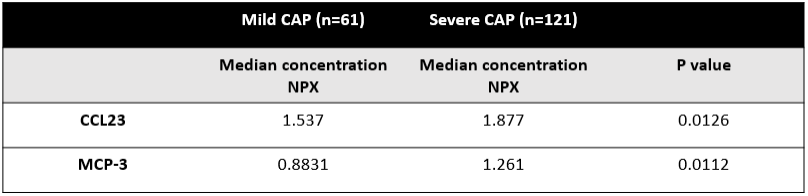

**NMC:**

nasal mucosal cytokine; CAP: community acquired pneumonia; NPX: normalized protein expression, arbitrary unit used in Olink assay that is log 2 scale. Mann-Whitney test was used to determine differences between mild and severe pneumonia

Quantification of NMC in children with CAP based on pathogen classification

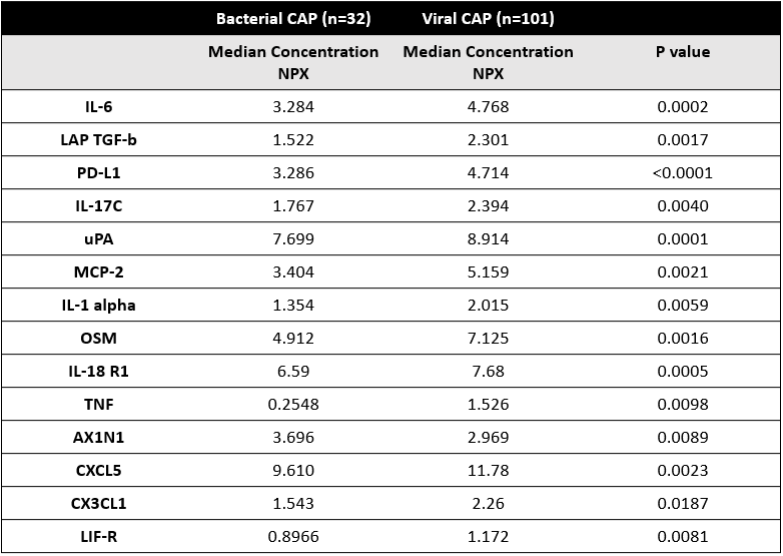

NMC: nasal mucosal cytokine; CAP: community acquired pneumonia; NPX: normalized protein expression, arbitrary unit used in Olink assay that is log 2 scale. Mann-Whitney test was used to determine differences between bacterial CAP and viral CAP.

**Conclusion:**

Children with severe CAP had higher monocyte chemoattractant NMC conc. than children with mild disease. Children with viral CAP, particularly influenza, had a more robust mucosal response including both proinflammatory and antiviral NMC than children with bacterial CAP. These findings show differences in NMC conc. based on etiology and disease severity. Further studies are needed to determine whether NMC are reliable predictive biomarkers of CAP etiology and severity.

**Disclosures:**

**Lilliam Ambroggio, PhD, MPH**, **Pfizer Inc** (Grant/Research Support) **Asuncion Mejias, MD, PhD, MsCS**, **Janssen** (Grant/Research Support, Advisor or Review Panel member)**Merck** (Grant/Research Support, Advisor or Review Panel member)**Roche** (Advisor or Review Panel member)**Sanofi** (Advisor or Review Panel member) **Octavio Ramilo, MD**, **Adagio** (Consultant)**Bill & Melinda Gates Foundation** (Grant/Research Support)**Janssen** (Grant/Research Support)**Lilly** (Consultant)**Merck** (Consultant, Grant/Research Support)**NIH** (Grant/Research Support)**Pfizer** (Consultant)**SANOFI** (Board Member)

